# Molecular Confirmation of Vancomycin-Resistant *Staphylococcus aureus* with *vanA* Gene from a Hospital in Kathmandu

**DOI:** 10.1155/2021/3847347

**Published:** 2021-12-02

**Authors:** Meera Maharjan, Anil Kumar Sah, Susil Pyakurel, Sabita Thapa, Susan Maharjan, Nabaraj Adhikari, Komal Raj Rijal, Prakash Ghimire, Upendra Thapa Shrestha

**Affiliations:** ^1^Department of Microbiology, Kantipur College of Medical Science, Sitapaila, Kathmandu, Nepal; ^2^Department of Microbiology, Annapurna Neurological Institute and Allied Sciences, Maitighar, Kathmandu, Nepal; ^3^Department of Microbiology, Shi-Gan International College of Science and Technology, Kathmandu, Nepal; ^4^Central Department of Microbiology, Tribhuvan University, Kirtipur, Nepal

## Abstract

*Staphylococcus aureus*, a commensal on the skin and in the nasal cavity of humans, is one of the most serious cases of nosocomial infections. Moreover, methicillin-resistant *S. aureus* (MRSA) is a leading cause of morbidity and mortality worldwide. For the treatment of MRSA infections, vancomycin is considered as a drug of choice. However, the emergence of vancomycin resistance among MRSA isolates has been perceived as a formidable threat in therapeutic management. To estimate the rate of vancomycin-resistant *S. aureus* (VRSA) and to detect the vancomycin-resistant genes, namely, *vanA* and *vanB*, among the isolates, a hospital-based cross-sectional study was conducted from July to December 2018 in Annapurna Neurological Institute and Allied Science, Kathmandu, Nepal. *S. aureus* was isolated and identified from different clinical samples and processed for antibiotic susceptibility testing by the modified Kirby–Bauer disc diffusion method. The screening of MRSA was performed as per Clinical and Laboratory Standard Institute (CLSI) guidelines. VRSA was confirmed by the minimum inhibitory concentration (MIC) method by employing E-test strips. All the phenotypically confirmed VRSA were further processed to detect the *vanA* and *vanB* gene by using the conventional polymerase chain reaction (PCR) method. A total of 74 (20.3%) *S. aureus* were isolated, and the highest percentage of *S. aureus* was from the wound samples (36.5%). Of 74 *S. aureus* isolates, the highest number (89.2%) was resistant to penicillin, and on the other hand, linezolid was found to be an effective drug. Likewise, 45 (60.81%) were found to be MRSA, five (11.11%) were VRSA, and 93.2% of *S. aureus* isolates showed an MAR index greater than 0.2. Two VRSA isolates (40%) were positive for the *vanA* gene. The higher prevalence of MRSA and significant rate of VRSA in this study recommend routine surveillance for the MRSA and VRSA in hospital settings before empirical therapy.

## 1. Introduction

Staphylococci are the most commonly isolated organisms, accounting for almost 30% of all hospital-acquired infections and 50% of bloodstream infections [1, 2], and have become a major public health threat as a result of increased drug resistance in this organism [3]. Staphylococcal resistance to antimicrobial agents is a worldwide concern with a history of almost 70 years. Penicillin was an effective drug to treat *Staphylococcus aureus* until the emergence of methicillin-resistant *S. aureus* (MRSA) in England in 1961 [4]; since then, MRSA has been reported as a leading cause of nosocomial infections [5]. Vancomycin is a choice of drug for the treatment of MRSA infections, which has resulted in the development of vancomycin-intermediate *S. aureus* (VISA) and vancomycin-resistant *S. aureus* (VRSA). In May 1996, the first VISA was reported in Japan [6]. Since then, VRSA strains were isolated in USA, Australia, Europe, and other Asian countries [7]. This resistance might be acquired by both mutation and/or the horizontal transfer of vancomycin-resistant genes among the bacteria [8]. Till now, 11 *van* genes, *vanA*, *vanB, vanD, vanF, vanI, vanM, vanC, vanE, vanG, vanL*, and *vanN*, have been described [9]. Among them, *vanA* and *vanB* genes are the most important and commonly observed in hospital isolates. The *vanA* gene is highly resistant to vancomycin and teicoplanin antibiotics, while the *vanB* gene shows higher resistance to vancomycin and susceptibility to teicoplanin [10]. Although VRSA remains rare, there is a global concern that VRSA poses by far the risk to the patient due to its virulent nature. This virulent nature of the organism with limited treatment options makes VRSA a global public threat [11]. According to the WHO, the pathogenicity and resistance patterns of *S. aureus* pose a great threat to global health. In addition, the resistant strains of *S. aureus*, MRSA, VISA, and VRSA, are recently classified as bacteria of high priority with the potential to cause significantly devastating mortality globally if adequate treatment options are not developed [12].

In Nepal, the prevalence of MRSA showed an increasing trend: 29.1%–68% [13–15]. On the other hand, comparatively few works on the detection of VISA, VRSA, and their encoding genes have been conducted [15, 16]. Therefore, the present study was aimed to detect the VRSA and their encoding genes, namely, *vanA* and *vanB*, among VRSA isolates obtained from different clinical specimens. In addition, the information obtained from our study can be utilized to formulate national antibiotic policy and appropriate control measures for further spread of VRSA.

## 2. Materials and Methods

### 2.1. Study Design

A hospital-based cross-sectional study was conducted among patients of Annapurna Neurological Institute and Allied Sciences, Kathmandu, Nepal, from July to Dec 2018. The study was approved by the Nepal Health and Research Council (NHRC). The sample size was calculated by using Fisher's formula. Patients of all age groups and sexes visiting the hospital during the study period who gave written consent to be enrolled were included for data and specimen collections. A total of 795 clinical samples were collected including urine (*n* = 279), sputum (*n* = 126), blood (*n* = 118), pus (*n* = 109), wound swab (*n* = 92), and others (*n* = 71; catheter tips, cerebrospinal fluid (CSF), synovial fluid, and ventriculoperitoneal shunt tips). Those samples showing the possible signs of contaminations were excluded from the study.

### 2.2. Sample Collection

Samples were collected according to standard laboratory protocols [17]. All valid specimens were cultured on suitable culture media as per their requirements.

### 2.3. Identification of *S. aureus*

Gram-positive organisms with grape-like clusters on microscopy and yellow colonies on mannitol salt agar (MSA) were primarily screened as *S. aureus* and were confirmed by performing different biochemical tests including catalase, coagulase, and oxidase tests [17, 18]. All the isolates confirmed as *S. aureus* were used for further analysis.

### 2.4. Antimicrobial Susceptibility Testing (AST)

All the identified *S. aureus* isolates were subjected to an *in vitro* antimicrobial susceptibility test by employing the modified Kirby–Bauer disc diffusion method as recommended by Clinical and Laboratory Standard Institute (CLSI) guidelines [19]. The antimicrobial disks used were ampicillin (30 *μ*g), cefoxitin (30 *μ*g), chloramphenicol (30 *μ*g), ciprofloxacin (25 *μ*g), clindamycin (31 *μ*g), cotrimoxazole (25 *μ*g), erythromycin (26 *μ*g), gentamycin (28 *μ*g), linezolid (30 *μ*g), oxacillin (10 *μ*g), tetracycline (20 *μ*g), and penicillin (10 *μ*g) from HiMedia, India.

### 2.5. Screening of MRSA and VRSA

*S. aureus* with a zone of inhibition size of 21 mm or less with a cefoxitin (CX30 *μ*g) disc was screened as MRSA and having a zone size of 16 mm or less with vancomycin (30 *μ*g) was considered as VRSA as per CLSI guidelines [19].

### 2.6. Confirmation of VRSA by MIC Using Vancomycin Strips (E-Test)

Carpet cultures of bacterial suspension were performed over the Mueller Hinton agar (MHA) plate. The E-test strip (HiMedia) was placed on an MHA plate with an MIC scale facing upward and the concentration maximum nearest to the rim of the plate. Then, the plates were incubated for 24 h at 37°C, and the zone of inhibition was measured [20]. The isolates were interpreted as vancomycin-sensitive *S. aureus* (VSSA), VISA, and VRSA if the size of zone of inhibition was ≥20 mm, 17–19 mm, and ≤16 mm, respectively. VRSA isolates were preserved in 20% glycerol containing tryptic soya broth and kept at −70°C until subsequent molecular tests were performed [21].

### 2.7. Molecular Detection of *vanA* and *vanB* Gene

The extraction of DNA (plasmid) of VRSA isolates was carried out by using the lysostaphin lysis method [22]. Genes encoding the vancomycin resistance determinants, *vanA* and *vanB*, were detected by conventional polymerase chain reaction (PCR) (Proflex, Thermo Fisher, USA) using specific primers manufactured by Macrogen Korea. The primers used were *vanA* F-GGCAAGTCAGGTGAAGATG, *vanA* R-ATCAAGCGGTCAATCAGTTC, *vanB* F-GTGACAAACCGGAGGCGAGGA, and *vanB* R- CCGCCATCCTCCTGCAAAAAA, respectively [23, 24]. A total of 25 *μ*l PCR reaction mixture was used for amplification of each gene. The PCR amplification of the *vanA* gene was carried out at an initial denaturation for 2 min at 94°C followed by 35 cycles of denaturation at 94°C for 1 min, annealing at 54°C for 1 min, and extension at 72°C for 1 min. The final extension had been performed at 72°C for 10 mins. Likewise, the *vanB* gene was amplified at an initial denaturation at 94°C for 10 min followed by 30 cycles of denaturation at 94°C for 30 sec, annealing at 50°C for 45 sec, and extension at 72°C for 30 sec. The final extension was performed at 72°C for 6 min. The obtained PCR products were electrophoresed in a 1.5% agarose gel which was stained with ethidium bromide (0.3 *μ*g/ml), visualized by using a UV transilluminator [25]. The molecular weight of the amplified product was estimated using a 100 bp DNA ladder (Solis Biodyne, Estonia).

### 2.8. Quality Control

All the tests were carried out by following standard aseptic techniques and CLSI guidelines. *S. aureus* (ATCC 25923) was used to ensure the performance of newly prepared media and the quality control of the AST and E-test. *Enterococcus fecalis* ATCC 51299 was used as a vancomycin-resistant control strain for molecular detection of resistant genes.

### 2.9. Statistical Analysis

All the data were entered and analyzed by using Statistical Package for Social Science (SPSS) version 24.

## 3. Results

### 3.1. Distribution of *S. aureus*

Of the 795 samples processed, 45.8% (364/795) showed growth containing 42.3% (154/364) Gram-positive bacteria. A total of 74 *S. aureus* isolates were obtained from the different clinical samples. The highest percentage (47.4%) was obtained from the wound swab followed by pus (41.0%). Likewise, the maximum percentage of isolates was obtained among the patients from the ICU (26.2%) followed by the Gynae ward (20.8%). A higher number of female patients were found to be infected than male patients (Table 1).

### 3.2. Antimicrobial Susceptibility Testing of *S. aureus*

Out of 12 different antimicrobial drugs used for AST, linezolid was found to be the most effective antimicrobial drug with 97.3% effectiveness to *S. aureus,* followed by clindamycin (36.5%) and gentamycin (36.5%). The highest resistance was found to penicillin (89.2%) and ampicillin (72.9%). Out of 74 *S. aureus*, 45 (60.8%) were found to be MDR (Table 2).

### 3.3. Antimicrobial-Resistant Phenotypes and Antimicrobial Resistance (MAR) Index

The highest number of isolates (35.1%) showed resistance to 11 antimicrobial drugs out of 12 except for linezolid followed by 12.2% isolates showing resistance to 10 antimicrobials. Only one isolate was found to be sensitive to all antimicrobial drugs. Altogether, 30 different types of resistant phenotypes were observed in our study among 74 isolates (Table 3).

Likewise, 93.2% of *S. aureus* isolates achieved an MAR index greater than 0.2 (Table 4).

### 3.4. Detection of the *vanA* Gene

**Figure 1:**
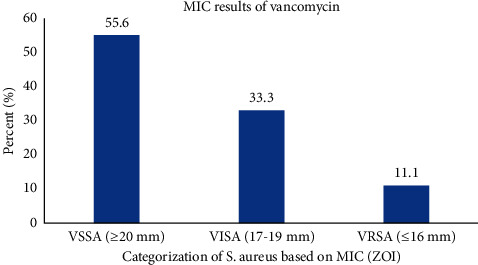
MIC result of vancomycin against MRSA isolates using an E-test strip (MIC; minimum inhibitory concentration, VSSA, vancomycin-sensitive *S. aureus*; VISA, vancomycin-intermediate *S. aureus*; VRSA, vancomycin-resistant *S. aureus*; and ZOI, zone of inhibition).

**Figure 2:**
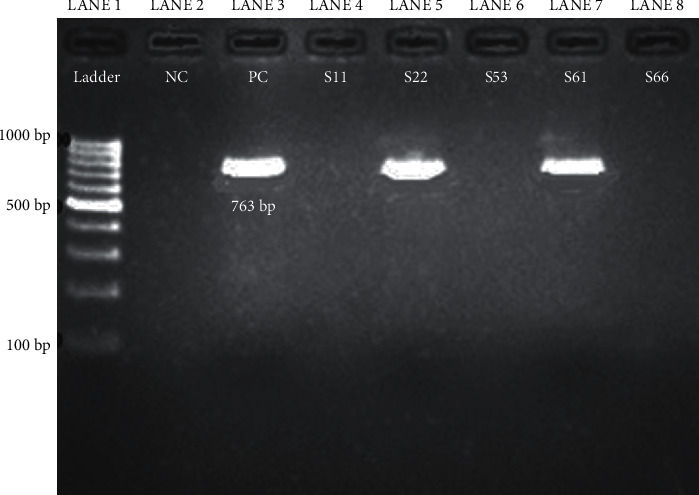
Amplification of the *vanA* gene from *S. aureus* isolates by conventional PCR (L1, DNA ladder 100–1000 bp; L2-NC, negative control; L3-PC, positive control; L4 to L8, *S. aureus* isolates; and S22 and S61 isolates were positive for the *vanA* gene).

Based on the results of the E-test for vancomycin among 45 MRSA isolates, the number VSSA, VISA, and VRSA was 25, 15, and 5, respectively (Figure 1). Of five VRSA-positive isolates, two isolates were found to be positive for the *vanA* gene whereas the *vanB* gene was not detected in this study (Table 5 and Figure 2).

## 4. Discussion

Vancomycin is the main therapeutic choice to treat MSRA. The early 1990s have shown a discernible increase in vancomycin use, and its excessive use for MRSA led to decreased susceptibility to vancomycin [26, 27]. However, in our study, we found 97.3% of *S. aureus* were sensitive to linezolid, so it can be the drug of choice for MRSA and VRSA strains. This study was in accordance with the work of Belbase et al. [28] which showed that all MRSA (100%) were sensitive to linezolid. On the other hand, most (89.2%) of the *S. aureus* isolates were resistant to penicillin. This result is similar to the previous works in Nepal [29, 30]. Of 74 *S. aureus* isolates, 45 (60.81%) isolates were MRSA. The finding of our study was consistent with other previous findings in Nepal [30–33]. However, the prevalence varies in the different parts of Nepal [34, 35] and India [36]. This finding can show the use of beta-lactam antibiotics against Gram-positive organisms in hospital and community settings. Although there are other antibiotics, including daptomycin and linezolid that may be effective against MRSA, increasing MRSA strains resistant to vancomycin have created a challenge in making clinical treatment decisions for such isolates [37]. Of 74 *S. aureus* isolates, we observed 30 different types of resistant phenotypes and 93.2% of *S. aureus* isolates achieved an MAR index greater than 0.2. This finding indicates the high-risk source of contamination due to continuous irrational use of antimicrobial drugs for empirical therapy in our hospital settings. Upon performing MIC, 5 (11.11%) of the MRSA isolates were reported as vancomycin-resistant strains which is higher than that in the study by Lama et al. [38] in Nepal and Thati et al. in India [39]. However, a higher prevalence (44.5%) of VRSA among MRSA isolates was reported in Nigeria [40]. Out of 5 VRSA, only 2 (40%) isolates showed *vanA* genes and no *vanB* genes were detected. In comparison to the study, the study performed by Saadat et al. [41] showed 40% of the isolates harbored at least one of the *van* genes. The remaining three isolates might possess other *van* genes rather than *vanA* and *vanB*, which were used in our study due to limitation of budget.

The predominant *S. aureus* (20.32%) out of 364 bacterial pathogens isolated in our study were confirmed by conventional phenotypic methods rather than the PCR method. As Nepal is a resource-limited country and most of the manpower have limited training, more advanced methods are used in some specific research activities only. Also, most of the clinical laboratories in hospitals including national-level ones rely on biochemical tests to identify organisms. By using similar methods, other different studies conducted in Nepal had reported *S. aureus* as the predominant bacterial pathogen [13, 33, 35, 42]; moreover, a high prevalence of *S. aureus* was also found in different parts of the world [43, 44]. This variation may be due to the time, type of samples, and the geographical, seasonal/climatic condition [44, 45]. The higher numbers of isolates (47.1%) were obtained from wound swab followed by pus (41%) and least from other samples (5.5%). Similarly, Kandel et al. [46] showed 41.03% of *S. aureus* obtained from wounds followed by urine (23.0%), pus (20.5%), and blood (10.3%), respectively. *S. aureus* is normally found in the environment and skin surface, so it is mostly obtained from wound swabs and pus.

Furthermore, once MRSA or VRSA resides in a hospital and environment, it is arduous to get rid of it, and the hospital environment may serve as a source of nosocomial infections in the future too [47]. Therefore, early detection of those sorts of resistance genes such as *van* genes would be a useful tool for the identification of infection and help in the prevention and control of their spread.

## 5. Conclusions

Detection of the *vanA* gene among MRSA isolates in our study alarms the spreading of the vancomycin-resistant gene among *S. aureus* and other bacterial pathogens. Moreover, the development of resistance to vancomycin, one of the few antibiotics available to treat MRSA infections, poses a serious problem in therapeutic management. However, linezolid could still be a choice of drug for the treatment of such infections.

## Figures and Tables

**Table 1:** Distribution of MRSA and VRSA isolates.

Organism/distribution	Growth positive, *n* (%)	Gram positive, *n* (%)	*S. aureus*, *n* (%)	MRSA, *n* (%)	VRSA, *n* (%)
Sex					
Male (*n* = 409)	215 (52.6)	93 (43.2)	42 (19.5)	29 (69.0)	3 (10.3)
Female (*n* = 386)	149 (38.6)	61 (40.9)	32 (21.5)	16 (50.0)	2 (12.5)
Clinical samples					
Urine (*n* = 279)	135 (48.3)	32 (23.7)	10 (7.4)	3 (50.0)	0
Sputum (*n* = 126)	61 (48.4)	29 (47.5)	13 (21.3)	5 (38.5)	0
Blood (*n* = 118)	20 (16.9)	7 (35.0)	6 (30.0)	2 (33.3)	0
Pus (*n* = 109)	54 (49.5)	34 (62.9)	16 (29.6)	14 (87.5)	3 (21.4)
Wound swab (*n* = 92)	57 (61.9)	42 (73.7)	27 (47.4)	21 (77.8)	2 (9.5)
^*∗*^Others (*n* = 71)	17 (23.9)	10 (58.8)	2 (11.8)	0	0
Hospital units					
General ward (*n* = 370)	160 (43.2)	66 (41.3)	32 (20.0)	20 (62.5)	2 (10.0)
OPD (*n* = 171)	91 (53.2)	35 (38.5)	15 (16.5)	9 (60.0)	1 (11.1)
ICU (*n* = 125)	65 (52.0)	31 (47.6)	17 (26.2)	12 (70.5)	2 (16.7)
Gynae (*n* = 129)	48 (37.2)	22 (45.8)	10 (20.8)	4 (40.0)	0
**Total (*n*** **=** **795)**	**364 (45.8)**	**154 (42.3)**	**74 (20.3)**	**45 (60.8)**	**5 (11.1)**

Abbreviations: MRSA = methicillin-resistant *Staphylococcus aureus*; VRSA = vancomycin-resistant *Staphylococcus aureus*; ^*∗*^others = throat swab, vaginal swab, synovial fluid, and pleural fluid; percentage calculated on respective row total.

**Table 2:** Antimicrobial susceptibility patterns of *S. aureus* isolates (*n* = 74).

Antimicrobial drugs	Sensitive, *n* (%)	Intermediate, *n* (%)	Resistant, *n* (%)
Ampicillin	11 (14.9)	9 (12.2)	54 (72.9)
Cefoxitin	29 (39.2)	0	45 (60.8)
Chloramphenicol	24 (32.4)	8 (10.8)	42 (56.8)
Ciprofloxacin	22 (29.7)	7 (9.5)	45 (60.8)
Clindamycin	27 (36.5)	6 (8.1)	41 (55.4)
Cotrimoxazole	22 (29.7)	5 (6.8)	47 (63.5)
Erythromycin	13 (13.6)	6 (8.1)	55 (74.3)
Gentamycin	27 (36.5)	5 (6.8)	42 (56.7)
Linezolid	72 (97.3)	0	2 (2.7)
Oxacillin	10 (13.5)	0	64 (86.5)
Penicillin	8 (10.8)	0	66 (89.2)
Tetracycline	18 (24.3)	2 (2.7)	54 (73.0)

**Table 3:** Antimicrobial-resistant phenotypes of *S. aureus* isolates (*n* = 74).

Resistant to no. of antimicrobial drugs	Resistant phenotypes	No. of isolates showing the pattern	Total no. of isolates (%)
12	AmpCxCCipCdCotEGenLzOxPTe	2	2 (2.7)

11	AmpCxCCipCdCotEGenOxPTe	26	26 (35.1)

10	AmpCxCipCdCotEGenOxPTe	1	9 (12.2)
	AmpCCipCdCotEGenOxPTe	8	

9	AmpCCipCotEGenOxPTe	1	4 (5.4)
	AmpCxCdCotEGenOxPTe	1	
	AmpCCipCdCotEGenOxTe	1	
	CxCCipCdCotEOxPTe	1	

8	CCipCdCotEGenOxTe	1	3 (4.0)
	AmpCCipEGenOxPTe	1	
	AmpCxCCipCotEOxTe	1	

7	CCipCdCotEOxP	2	10 (13.5)
	CxCCipCdCotEOx	3	
	AmpCxEGenOxPTe	4	
	AmpCipCdCotEOxP	1	

6	AmpCxGenOxPTe	1	2 (2.7)
	CCipCdCotEOxTe	1	

5	CCipCotEPTe	1	5 (6.8)
	AmpCxOxPTe	1	
	AmpEOxPTe	2	
	AmpCotEOxP	1	

4	AmpCCipP	1	5 (6.8)
	AmpEOxP	1	
	AmpCxOxP	1	
	AmpEPTe	1	
	AmpCxPTe	1	

3	AmpOxP	2	3 (4.0)
	AmpCxP	1	

2	AmpP	3	3 (4.0)

1	P	1	1 (1.4)

Sensitive to antimicrobial drugs		1	1 (1.4)

**Total**		**74**	**74 (100)**

Amp, ampicillin; Cx, cefoxitin; C, chloramphenicol; Cip, ciprofloxacin; Cd, clindamycin; Cot, cotrimoxazole; E, erythromycin; Gen, gentamycin; Lz, linezolid; Ox, oxacillin; P, penicillin; Te, tetracycline (isolates intermediately resistant were included in the resistant category to determine their antimicrobial-resistant phenotypes).

**Table 4:** Antimicrobial resistance (MAR) index of *S. aureus* isolates (*n* = 74).

MAR indices	No. of *S. aureus* isolates (%)
0	1 (1.4)
0.08	1 (1.4)
0.17	3 (4.0)
0.25	3 (4.0)
0.33	5 (6.8)
0.42	5 (6.8)
0.50	2 (2.7)
0.58	10 (13.5)
0.67	3 (4.0)
0.75	4 (5.4)
0.83	9 (12.2)
0.92	26 (35.1)
1	2 (2.7)
**Total**	**74**

**Table 5:** Molecular screening of VRSA for *vanA* and *vanB* genes (*n* = 5).

MSRA	Screening of VRSA, *n* (%)	Confirmation of VRSA by tde E-test, *n* (%)	Detection of VRSA encoding genes	
			*vanA*, *n* (%)	*vanB*, *n* (%)

45	9 (20.0)	5 (11.1)	2 (40.0)	0

## Data Availability

The datasets generated and analyzed will be available on request to the corresponding author.

## Additional Points

The study was conducted in a single setting for six months with 795 samples. So, the generalization of the findings may not be accurate. In addition, this study was a self-funded research, and we did not have enough funds to detect all *van* genes among MRSA isolates. However, based on the literature review, VRSA isolates, which harbor *vanA and vanB* genes, were the most common phenotypes, so we did focus on these two genes.
